# Organische Arsenverbindungen und ihre Chemotherapeutische Bedeutung

**Published:** 1913-03

**Authors:** 


**Organische Arsenverbindungen und ihre Chemotherapeutische Bedeutung.** Von Doz. Dr. M. NIERENSTEIN. Pp. 94. Stuttgart: Ferdinand Enke. 1912.

This important paper on the therapeutic properties of the organic arsenic compounds appeared in Volume XIX of the *Sammlung Chemischer und Chemischer-technischer Vorträge*, edited by Professor Herz of Breslau. Its author, Dr. Nierenstein, is now a member of the staff of the University of Bristol, and the selection of this contribution for publication by Professor Herz sets the seal of European approval upon the work. Without criticising the conclusions, we may express the interest with which we have read the work, and our appreciation of the supreme importance to medicine of these advances in bio-chemical knowledge.

The section dealing with the history of the therapeutic use of arsenic covers a period beginning with Dioscorides, and includes names such as Paracelsus, Fowler, Cadet,

Bunsen, and, in our own times, Ehrlich, and many more. The modern discovery of the diminished toxicity of certain organic arsenical compounds dates from Bunsen's work on cacodylic acid, which is readily soluble, and in spite of its high arsenic content shows little or no toxicity. The arsenic here appears to be combined in a manner different from that of its inorganic compounds. Ehrlich set himself the task of explaining the precise nature of this difference, and determined that the non-toxicity of the arsenic in cacodyl depended upon its pentavalency, for in this condition it lost its activity towards protoplasm. He then endeavoured to obtain organic arsenic preparations in which the arsenic is trivalent, and therefore active as regards pathogenic protozoa. The result was the discovery of such compounds as arseno-phenylglycin and dioxydiaminoarsenobenzol (salvarsan). Dr. Nierenstein briefly reviews the various cacodylates which were obtained by Bunsen and Bäyer, and goes on to demonstrate the progressive advances made until the relatively non-toxic group of "arrhenal" compounds was produced from methyl-arsenate. Passing on to combinations with the higher carbon series, the aromatic series of arsenic compounds were formed, which contained phenyl, di-phenyl or tri-phenyl groups, and are known accordingly as the primary, secondary, and tertiary aromatic arsines. Of these the "secondary" series are highly toxic. Nierenstein, working with Moore and Todd in Liverpool, found that so far as trypanosomes were concerned the cacodylic and arrhenal compounds were inert. Then "atoxyl" was arrived at (the sodium salt of p-amino-phenylarsonate), a drug well known in medicine. The experimental work of Nierenstein and others has already been published in the *Sleeping Sickness Bulletin* of the Tropical Diseases Bureau, Imperial Institute, London, and is not referred to at length in this paper.

The remainder of the article is devoted to a discussion of the theories of the action of atoxyl. These theories are three in number: Ehrlich's *Reduction Theory*, Breinl and Nierenstein's *Oxidation Theory*, and Uhlenhuth's *Partial Cell-action Theory*. Here we are introduced to bio-chemical phenomena of absorbing interest. The argument turns largely upon the activity of the arylarsonates against protozoa in the living blood-stream, as contrasted with their inactivity against protozoa *in vitro*. Ehrlich and Hata suggest "that spirochætes possess a whole series of chemo-receptors: only those whose affinity for the corresponding atom-groups of the chemicals is considerably stronger than that of the host's tissues are of any account in chemotherapy. These receptors may include an *arseno-ceptor*, an *oxyamido-ceptor*, and a *halogen-ceptor*, whose active affinity for dioxydiamidoarsenobenzol may explain the ready destruction of the parasites, without injury to the host.

Ehrlich conjectures that in the protoplasm of the trypanosome arseno-ceptors exist, with a special affinity for toxic reduction-products formed by the host's tissues from the non-toxic arylarsonate.

Breinl and Nierenstein do not accept Ehrlich's view of a reduction process taking place, but explain the therapeutic action of atoxyl as follows :—

(1) That the amido group of atoxyl unites with serum-proteid to form a so-called "atoxyl-serum."

(2) That by an oxidation process (possibly due to ferments) the "atoxyl-serum" is oxydised, and arsenic set free.

(3) That simultaneously a reduction takes place, splitting the atoxyl into arsenious acid and aniline, the aniline being excreted in the fæces.

(4) That the arsenic, liberated partly by oxidation, partly by reduction ferments, and probably also by the trypanosomes themselves, exerts in a nascent state a destructive effect on the parasites.

These authors think that the amino group is the anchoring-group which binds the atoxyl to the serum. They have separated "atoxyl serum," and liberated the arsenic with liver emulsion.

Uhlenhuth's theory is that the action of atoxyl is an indirect one, which by stimulating the cells of the host causes them to manufacture some substance antagonistic to the parasites.

Dr. Nierenstein carefully examines these various theories, and quotes a great deal of chemical and bio-chemical experiment to elucidate the problems involved. It is a fine, clear piece of work, and the reasoning is able and direct. There is evidence of tremendous energy in the experimental investigations which he has himself conducted, and we congratulate him on the readable form in which he has handled this abstruse subject. Medical science in Bristol cannot fail to profit by the presence of such workers in the University.

